# Supplementary figures and images for: Comparison between enzyme-linked immunospot assay and intracellular cytokine flow cytometry assay of cytomegalovirus-specific T-cell response in healthy participants
Source: PLoS One. 2026 Jun 4;21(6):e0349292. doi: 10.1371/journal.pone.0349292 (PMC13235872; doi:10.1371/journal.pone.0349292)

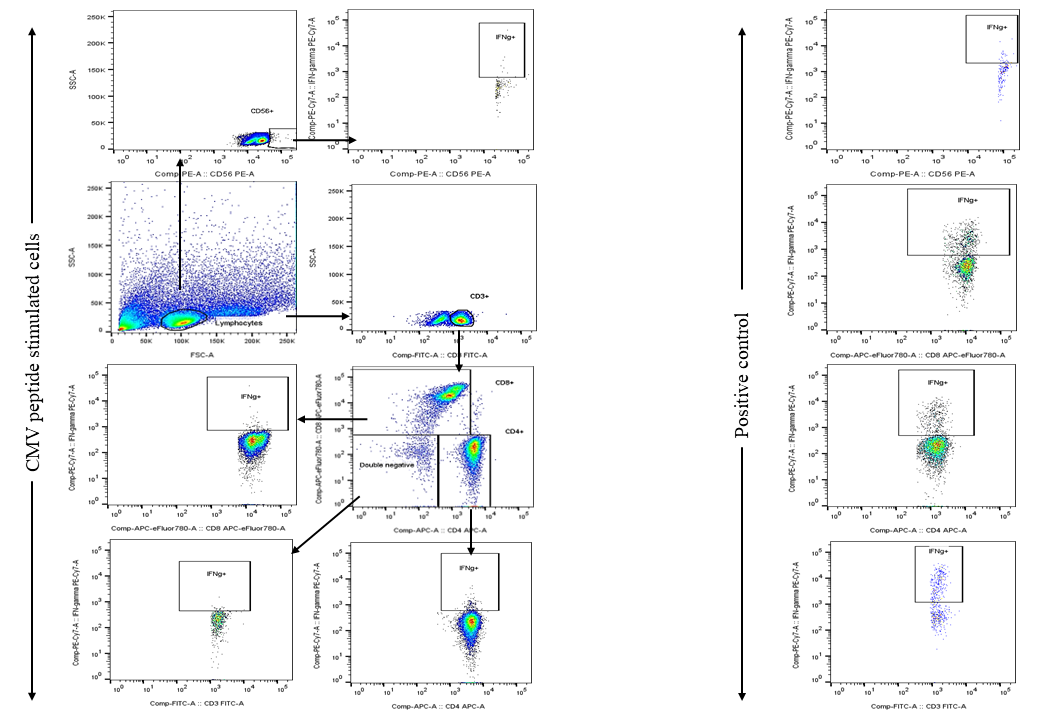

Supplement: S1 Fig — (TIF) [file pone.0349292.s001.tif]
