# Supplementary material for: Comparison between enzyme-linked immunospot assay and intracellular cytokine flow cytometry assay of cytomegalovirus-specific T-cell response in healthy participants
Source: PLoS One. 2026 Jun 4;21(6):e0349292. doi: 10.1371/journal.pone.0349292 (PMC13235872; doi:10.1371/journal.pone.0349292)
Supplement: S1 Table — (DOCX) [file pone.0349292.s002.docx]

**Table S1. The percentage of IFN-γ producing cells of individual participants.**

| **No.** | **IFN-γ producing cells by ICS (% per each cell type)** | | | | | | | | | | | | | | |  |  |
| --- | --- | --- | --- | --- | --- | --- | --- | --- | --- | --- | --- | --- | --- | --- | --- | --- | --- |
|  | **Non-stimulated cells** | | | | | **wCMV-stimulated cells** | | | | | **IE1-stimulated cells** | | | | |  |  |
|  | **CD3** | **CD4** | **CD8** | **DN** | **NK** | **CD3** | **CD4** | **CD8** | **DN** | **NK** | **CD3** | **CD4** | **CD8** | **DN** | **NK** | |  |
| 1 | 0.027 | 0.068 | 0.157 | 0.260 | 4.511 | 0.148 | 0.522 | 0.466 | 1.048 | 17.391 | 0.046 | 0.241 | 0.177 | 0.578 | 0.000 | |  |
| 2 | 0.122 | 0.288 | 0.158 | 0.303 | 3.788 | 0.409 | 0.528 | 0.505 | 1.183 | 2.733 | 0.141 | 0.318 | 0.326 | 0.373 | 5.618 | |  |
| 3 | 0.032 | 0.149 | 0.086 | 0.219 | 3.947 | 0.055 | 0.178 | 0.203 | 0.646 | 6.024 | 0.087 | 0.175 | 0.150 | 0.921 | 3.636 | |  |
| 4 | 0.136 | 0.122 | 0.132 | 0.264 | 7.317 | 0.227 | 0.336 | 0.535 | 3.371 | 9.375 | 0.611 | 0.728 | 0.460 | 2.632 | 28.302 | |  |
| 5 | 0.087 | 0.085 | 0.107 | 0.582 | 3.922 | 0.537 | 0.369 | 0.763 | 1.345 | 6.154 | 0.258 | 0.171 | 0.535 | 1.124 | 5.263 | |  |
| 6 | 0.016 | 0.058 | 0.028 | 0.197 | 0.000 | 1.474 | 2.185 | 1.760 | 2.149 | 28.926 | 0.351 | 0.236 | 1.614 | 0.928 | 8.000 | |  |
| 7 | 0.025 | 0.069 | 0.090 | 0.309 | 5.128 | 0.264 | 0.449 | 0.219 | 0.402 | 11.765 | 0.375 | 0.502 | 0.556 | 1.317 | 7.692 | |  |
| 8 | 0.278 | 0.641 | 0.402 | 0.666 | 14.371 | 1.052 | 1.454 | 0.945 | 1.970 | 14.737 | 0.322 | 0.761 | 0.538 | 1.473 | 29.167 | |  |
| 9 | 0.240 | 0.303 | 0.296 | 0.640 | 2.326 | 0.339 | 0.536 | 0.439 | 0.728 | 9.231 | 0.435 | 0.855 | 1.406 | 0.781 | 29.167 | |  |
| 10 | 0.067 | 0.072 | 0.643 | 0.336 | 19.792 | 0.302 | 0.296 | 1.824 | 2.569 | 11.348 | 1.267 | 1.347 | 2.517 | 2.505 | 81.860 | |  |
| 11 | 0.021 | 0.037 | 0.088 | 0.122 | 2.326 | 0.048 | 0.199 | 0.309 | 1.250 | 9.231 | 0.045 | 0.174 | 0.260 | 1.299 | 10.294 | |  |
| 12 | 0.017 | 0.022 | 0.051 | 0.111 | 1.887 | 0.130 | 0.176 | 0.432 | 0.497 | 7.937 | 0.049 | 0.061 | 0.323 | 0.366 | 5.714 | |  |
| 13 | 0.023 | 0.010 | 0.027 | 0.092 | 0.000 | 0.354 | 0.430 | 0.475 | 0.536 | 4.130 | 0.079 | 0.049 | 0.119 | 0.240 | 5.769 | |  |
| 14 | 0.016 | 0.028 | 0.048 | 0.436 | 2.564 | 0.152 | 0.213 | 0.578 | 1.805 | 3.125 | 0.183 | 0.220 | 0.504 | 1.255 | 4.673 | |  |
| 15 | 0.016 | 0.024 | 0.014 | 0.125 | 1.370 | 0.129 | 0.133 | 0.163 | 0.350 | 16.822 | 0.112 | 0.156 | 0.185 | 0.812 | 5.882 | |  |
| 16 | 0.018 | 0.119 | 0.075 | 0.246 | 3.070 | 0.414 | 0.761 | 0.542 | 1.923 | 16.447 | 0.284 | 0.383 | 0.383 | 1.459 | 21.053 | |  |
| 17 | 0.012 | 0.033 | 0.074 | 0.160 | 2.586 | 0.141 | 0.130 | 0.106 | 0.586 | 6.667 | 0.113 | 0.385 | 0.245 | 0.911 | 9.589 | |  |
| 18 | 0.018 | 0.054 | 0.059 | 0.248 | 1.923 | 0.097 | 0.146 | 0.137 | 1.446 | 4.151 | 0.133 | 0.094 | 0.245 | 0.674 | 8.955 | |  |
| 19 | 0.018 | 0.049 | 0.058 | 0.025 | 5.797 | 0.089 | 0.203 | 0.179 | 0.367 | 12.963 | 0.078 | 0.103 | 0.174 | 0.200 | 8.861 | |  |
| 20 | 0.043 | 0.103 | 0.060 | 0.421 | 0.000 | 4.668 | 3.054 | 4.548 | 5.162 | 1.937 | 1.013 | 1.715 | 1.193 | 2.595 | 6.944 | |  |
| 21 | 0.040 | 0.024 | 0.047 | 0.404 | 0.621 | 0.160 | 0.056 | 0.202 | 1.234 | 1.796 | 0.100 | 0.048 | 0.189 | 1.014 | 3.243 | |  |
| 22 | 0.457 | 0.454 | 0.524 | 0.869 | 71.574 | 2.254 | 2.035 | 2.125 | 3.682 | 85.930 | 0.109 | 0.151 | 0.333 | 1.131 | 45.238 | |  |
| 23 | 1.036 | 0.892 | 1.017 | 1.062 | 0.000 | 0.600 | 0.856 | 1.464 | 1.956 | 10.606 | 1.436 | 1.627 | 1.795 | 2.232 | 3.158 | |  |
| 24 | 0.294 | 0.363 | 0.218 | 0.488 | 3.093 | 1.861 | 1.210 | 2.812 | 5.342 | 54.098 | 0.335 | 0.641 | 0.739 | 0.850 | 7.609 | |  |
| 25 | 0.156 | 0.123 | 0.210 | 0.534 | 3.125 | 0.412 | 0.577 | 0.411 | 2.332 | 10.577 | 0.251 | 0.402 | 0.182 | 1.295 | 10.667 | |  |
| 26 | 0.285 | 0.157 | 0.660 | 0.620 | 11.024 | 0.595 | 0.295 | 1.548 | 1.027 | 4.525 | 0.378 | 0.257 | 1.043 | 0.733 | 11.905 | |  |
| 27 | 0.082 | 0.112 | 0.230 | 0.087 | 0.000 | 2.037 | 2.035 | 2.306 | 8.854 | 52.885 | 0.416 | 0.682 | 1.225 | 2.700 | 0.000 | |  |
| 28 | 0.113 | 0.137 | 0.256 | 0.317 | 1.105 | 0.224 | 0.201 | 0.496 | 0.963 | 2.295 | 0.247 | 0.273 | 0.457 | 0.895 | 3.475 | |  |
| 29 | 0.083 | 0.089 | 0.038 | 0.204 | 4.206 | 0.760 | 0.621 | 1.593 | 1.457 | 10.864 | 0.715 | 0.633 | 1.404 | 1.231 | 13.916 | |  |
| 30 | 0.047 | 0.060 | 0.068 | 0.093 | 3.077 | 0.120 | 0.424 | 0.640 | 0.020 | 6.667 | 0.315 | 0.700 | 0.433 | 0.316 | 6.965 | |  |
