# Supplementary material for: Comparison between enzyme-linked immunospot assay and intracellular cytokine flow cytometry assay of cytomegalovirus-specific T-cell response in healthy participants
Source: PLoS One. 2026 Jun 4;21(6):e0349292. doi: 10.1371/journal.pone.0349292 (PMC13235872; doi:10.1371/journal.pone.0349292)
Supplement: Attachment — Submitted filename: Response to reviewers.docx [file pone.0349292.s004.docx]

Response to reviewers

**Reviewer#1:**

Cell-mediated immune response (CMIR) plays an important role in controlling CMV replication. Monitoring CMIR against CMV is crucial in clinical settings involving immunocompromised patients who are vulnerable to CMV infection or latent reactivation. In this study, the authors compared 2 detection methods of CMIR against CMV in peripheral blood mononuclear cells (PBMCs) from 30 healthy donors: 1). Intracellular staining (ICS) to quantify CMV-specific IFN-g production by T-cell subsets (CD3, CD4, CD8, double negative ND T cells) and 2). Enzyme-linked immunospot (ELISPOT) to quantify CMV-specific IFN-g secreting cells. Both whole CMV lysates and a peptide pool from the CMV IE-I protein were used to stimulate the PBMCs. They found that CMV-specific CD3 T cell responses as measured by ICS correlated well with IFN-g secreting cells quantified by ELISPOT. They also found that the anti-CMV IgG level correlated with the number of IE1-specific IFN-g producing cells.

Overall, the manuscript is well written, and the results generally supported the main conclusions. However, the authors need to be precise in their description of CMV-specific T cell response as measured by ICS and ELISPOT assays. While it is true for ICS, ELISPOT assay is not able to reveal the exact cytokine-producing cell types. Even though IFN-γ-producing cells within the PBMC are mainly CD3+ cells, other cell types such as NK cells can also contribute. In addition, the following issues also need to be addressed/clarified.

1. The p values for the correlations in the Abstract were missing.

We would like to thank you for your kind suggestion. The *p* values corresponding to the reported correlations have now been added to the Abstract at lines 35 – 37.

2. Line 38 in The Abstract stated there were 30 CMV-seropositive participants. Should this be 28?

We thank the reviewer for pointing out this important issue. The original wording in line 38 stated that “Among CMV-seropositive participants, thirty (100%) had CMV-specific T cell responses,” which may have caused confusion. Although 28 of the 30 participants (93.3%) were CMV-seropositive, CMV-specific T cell were detected in all participants (100%), including the two seronegative individuals. (Page 2, Lines 37-39)

“Among the 30 participants, twenty-eight (93.3%) were CMV-seropositive, but CMV-specific T-cells were detected in all participants (100%), including the two seronegative individuals.”

3. Table 1: What is relevance of BMI for this study? Does BMI correlate with cytokine production?

We thank the reviewer for this insightful comment. The primary objective of this study was to compare CMV-specific IFN-γ–secreting cells measured by ELISPOT with those quantified by intracellular cytokine staining (ICS). BMI was included in Table 1 as a baseline demographic characteristic to describe the general health status of the study population and to exclude potential confounding metabolic or inflammatory conditions.

In our cohort, the mean BMI was 23.09 kg/m², which falls within the normal range, and none of the participants were obese. In addition, all participants had no history of immune-related diseases, active viral infections, or other underlying medical conditions, according to the inclusion criteria. Therefore, no formal correlation analysis between BMI and cytokine production was performed. The limited BMI variability and absence of obesity in this cohort were unlikely to influence IFN-γ responses.

Previous studies have demonstrated that obesity can be associated with altered cytokine production and immune dysregulation; however, this effect is mainly observed in obese populations rather than individuals with normal BMI. Accordingly, BMI in the present study served to confirm cohort homogeneity rather than as a variable for immunological outcome analysis. (References. <https://doi.org/10.1016/bs.acc.2020.06.004>, <https://doi.org/10.1371/journal.pone.0121971>)

4. Please specify whether the NK-cells were gated on CD3-CD56+ or CD56+ lymphocyte population in the Fig 1 legend and the text. NK cells (CD3-CD56+) usually composed of about 5% to 10% of circulating lymphocytes. Why NK cells represented only 0.2% of total lymphocytes as indicated in Table 2?

We sincerely thank the reviewer for raising this important and insightful point. We fully agree that natural killer (NK) cells typically account for approximately 10–20% of total lymphocytes in peripheral blood. The relatively lower proportion of NK cells observed in the present study is most likely attributable to a technical limitation related to the sample preparation protocol. Specifically, cells were fixed and permeabilized prior to antibody staining, a procedure that may alter antigenic epitopes and consequently reduce the sensitivity of CD56 antibody detection. This effect may have led to an underestimation of the NK cell population. In support of this explanation, we have previously reported NK cell staining performed without formaldehyde fixation, in which the proportion of NK cells in the adult population was approximately 15%, consistent with established literature. (Srisala S, et al. BMC Res Notes 2019; 12: 633). To ensure transparency and appropriate interpretation of our findings, we have now explicitly addressed this issue in the Discussion section as a study limitation. The following sentence has been added to the manuscript at page 17, lines 340 – 343:

“Finally, the percentage of NK cells reported in this study may be underestimated, as cell fixation and permeabilization were performed prior to antibody staining, a process that could alter the recognized epitopes of CD56 and reduce staining efficiency.”

5. Lines 175-177 mixed the function of ICS and ELISPOT assays. ICS, not ELISPOT, quantifies IFN-g-producing T cells. ICS quantifies cytokine-producing cells, not cytokine-secreting cells. Please be precise in the description in Table 2 and in the text.

We agree with the reviewer on this important point. We have revised the text to clearly distinguish the functions of ICS and ELISPOT assays. Specifically, ICS is now consistently described as quantifying IFN-γ–producing T cells, whereas ELISPOT is described as measuring IFN-γ–secreting cells without phenotypic discrimination. These corrections have been applied to Table 2 and throughout the manuscript to ensure precise and accurate terminology.

6. Table 3 shows that some subjects such as 9,12, 15 had a high background of IFN-g SFU. Was this background subtracted from the CMV antigen-specific IFN-g SFU? Was this high background IFN-g secretion correlated with high IFN-g production in unstimulated cells by ICS? It would be informative to add a supplemental table to show the % of IFN-g-producing T cells for individual untreated and CMV-stimulated samples.

We thank the reviewer for this detailed comment. CMV antigen–specific IFN-γ SFU values were calculated after subtraction of background signals from unstimulated control cells.

The high background IFN-γ secretion observed in the ELISPOT assay did not correlate with IFN-γ production measured by ICS in unstimulated cells. Among the three subjects with high background ELISPOT responses (Subjects No. 9, 12, and 15), only one subject showed concordant results between ELISPOT and ICS.

These discrepancies likely reflect inherent methodological differences between the two assays. ELISPOT measures cumulative IFN-γ secretion from all PBMCs immediately after stimulation, whereas ICS detects intracellular cytokine production within defined lymphocyte subsets. As a result, ELISPOT may capture cytokine secretion from additional cell types or early secretory events that are not detected by ICS.

In response to the reviewer’s suggestion, we have added a supplementary table (Supplementary Table S1) showing the percentage of IFN-γ–producing T cells for individual participants under unstimulated and CMV antigen-stimulated conditions.

7. Fig. S1. CD8 seemed to be under compensated against CD4 in the flow cytometric plot. Please double check flow compensations and show proper gating of NK cells and their cytokine production.

We thank the reviewer for raising this point. The flow cytometry data were already properly compensated during the initial analysis, and no changes to the compensation settings were applied. For clarification, we have included Figure 1 in this response to reviewers to illustrate the original compensation. The fluorescence-conjugated antibodies used were anti-CD4 APC and anti-CD8 APC/EF780, which are excited by the same red laser (633–640 nm) but have distinct emission peaks at approximately 660 nm and 780 nm, respectively. Figure 2 is provided to demonstrate the spectral properties of these fluorochromes. Some degree of signal spreading between these channels may still be observed due to spectral overlap, particularly following fixation and permeabilization.

Importantly, this phenomenon does not affect the gating strategy or interpretation of cytokine-producing T-cell subsets. To improve clarity, we have revised Figure S1 to explicitly show NK cell (CD3⁻ NK1.1⁺) gating and their IFN-γ production. These data confirm that the reported CMV-specific IFN-γ responses predominantly originate from CD3⁺ T cells rather than NK cells.

The issue of low percentage of NK cells in this study was addressed in item 4.

Fig 1. Flow cytometry compensation

Fig 2. Excitation and emission of APC and APC-EF780

8. Figure 4A shows the seronegative participant had a high number of SFU, but very few ICS spots. What is the reason for this discrepancy? Which 2 donors were seronegative?

We thank the reviewer for this important comment. The two seronegative participants were Subject No. 1 and Subject No. 30. Both individuals exhibited a relatively high number of IFN-γ spot-forming units (SFU) detected by ELISPOT, while showing low frequencies of IFN-γ–producing T cells measured by ICS.

This discrepancy is likely attributable to intrinsic differences between the two assays. ELISPOT measures cumulative IFN-γ secretion from all cell types within PBMCs immediately after stimulation, whereas ICS detects intracellular cytokine production within defined lymphocyte subsets following brefeldin A treatment. As a result, ELISPOT may capture IFN-γ secretion from non-T-cell populations or early secretory events that are not detected by ICS.

9. Lines 245-246 stated “Furthermore, a higher significance correlation was found in whole CMV stimulation than in CMV IE-1 peptide pool stimulation.”. Was this based on statistical analysis?

We thank the reviewer for this comment. The statement “a higher significance correlation was found in whole CMV stimulation than in CMV IE-1 peptide pool stimulation” was based on statistical analysis using Spearman’s rank correlation. The results showed that CMV-specific CD3⁺ T-cell responses measured by ICS were more strongly correlated with IFN-γ spot numbers obtained by ELISPOT following whole CMV stimulation than IE-1 peptide pool stimulation (wCMV: r = 0.7677, p < 0.0001; IE-1: r = 0.6516, p < 0.0001), as shown in Figures 3A and 3B.

10. Lines 278-280 stated “Stimulation of cells by peptide pools, IE1 in this study, is mediated by co-stimulation with MHC class I while whole CMV lysate is more likely to stimulate T-cells with MHC class II.” If IE-1 peptides are HLA class I epitopes, why there were IE-I specific IFN-g response in CD4 cells as well as NK cells? For Table 2, it could be very informative to add % of IFN-g+ unstimulated T cells and NK cells, especially considering the high background of IFN-g SFU.

We would like to thank the reviewer for highlighting this important point. We agree that CMV IE1 antigens can be recognized through both MCH class I and II restricted pathways. We would like to apologize for the oversight in our previous discussion. We have revised the manuscript by deleting these sentences from the discussion. “Stimulation of cells by peptide pools, IE1 in this study, is mediated by co-stimulation with MHC class I, while whole CMV lysate is more likely to stimulate T-cells with MHC class II. This implies that the magnitude of humoral immune response may better correlate with MHC class I stimulation and represent recent or current subclinical CMV reactivation.”

11. CD3 cells are composed mainly of CD4, CD8, and DN cells. Why there were lower number of IFN-g+ CD3 cells than IFN-g+ CD4, CD8, or DN?

We agree with the reviewer on this point. In this study, we reported the median number of IFN-γ–producing cells per 10⁶ cells for each T-cell subset following stimulation with whole CMV or the IE1 peptide pool, rather than expressing these values as proportions within the same parent population (CD3⁺ T cells). Consequently, the reported numbers of IFN-γ–producing CD4⁺, CD8⁺, and double-negative (DN) T-cell subsets are not directly additive and therefore do not necessarily equal the total number of IFN-γ–producing CD3⁺ T cells.

**Reviewer #2:**

The paper is well written, the methods have been rigorously performed and the results have been discussed critically. The authors have also listed the limitations of their study.

I have no further comments.

We sincerely appreciate the reviewer’s thorough and constructive review of our manuscript. We are grateful for your positive assessment. Your confirmation provides valuable validation of our research approach and presentation. We thank you for your time and professional evaluation.

**Reviewer #3:** Review for Klinmalai et al.

Summary

HCMV is a medically relevant pathogen with limited treatment options and very high seroprevalence, so work on its rapid and accurate diagnosis and clinical assessment of patient immune competence is urgently needed. Klinmalai et al. have carried out a quantitative comparison of two of the major methods for determining patient immune responses to HCMV. While the topic they address is not novel, the work appears strong and I would agree that regular re-evaluation of current protocols has value for patient outcomes.

The authors show a comparison of two well-established techniques for assessing immune effector functions: ELISPOT assay, which measures secreted cytokines; and intracellular cytokine staining (ICS), which measures cytokine-containing granules. Both measure only one target (interferon gamma, IFNg) and correlate strongly. While this is the expected outcome – as each of these protocols has been established for some time – it may be valuable to have this confirmatory study added to the body of literature on the topic.

The strength of the work lies in its simplicity, easily quantifiable outcomes, and statistical rigor. A clear positive/negative readout for IFNg-positive cells leaves little doubt as to the validity of each result. The authors use two stimulation techniques, either whole virus lysate or purified peptides, which further strengthen their data.

The paper does contain one very significant weakness, which is the absence of CMV-negative donors. If the authors are able to find CMV-negative donors to act as a negative control population, this work will be strengthened exponentially. Alternatively, given that certain geographic regions have seroprevalence approaching 100% and finding negative donors may be impossible, the authors should adequately justify how they validate their results without such controls.

Overall, I believe this paper has merit and rigor and acts to reinforce our understanding of commonly used diagnostic methods.

**Major points.**

1. The most important point, as mentioned above, is the lack of appropriate negative controls. Initially, the authors define their cohort as 30 patients, of whom 28 are CMV-seropositive and two -seronegative. However, as they acknowledge, both seronegative patients had a substantial population of HCMV-reactive T cells among their PBMCs. This indicates one of two things: either (A) their seropositivity is incorrect, and they in fact had only HCMV-positive donors in their cohort and no negative controls at all, which weakens the statistical relevance of the study; or (B) the reported seropositivity is correct, and the T cell assays show false-positive results, which weakens the physiological relevance and calls into question the validity of their assay results.

Either of these two cases is problematic for the authors’ conclusions. In case (A), the authors are attempting to publish work lacking adequate controls. This may simply reflect the unavoidable nature of HCMV – in certain demographics and geographic locales, HCMV seropositivity is nearly 100%. I suspect that’s the case in their region, but if so, the authors should adequately address this and explain how it impacts their results.

In case (B), the authors demonstrate nicely that both ELISPOT and ICS are prone to identical false positives. This might indicate a necessity for defining new thresholds positivity or might call into question the value of these methods entirely.

It’s essential that the authors explain to the reader why or why not each of these cases is a valid concern.

We thank the reviewer for raising the important issue of CMV-seronegative controls. We agree that the inclusion of CMV-negative donors would strengthen the study; however, CMV seroprevalence in our geographic region is exceptionally high, making the recruitment of truly CMV-naïve adult donors extremely challenging. This limitation has now been explicitly acknowledged and discussed in the revised manuscript.

“CMV-specific T-cell responses were detected by both ICS and ELISPOT assays in the two individuals classified as anti-CMV IgG seronegative, with relatively high response magnitudes. Importantly, this finding does not necessarily indicate assay false positivity or serological misclassification. Functional T-cell assays such as ELISPOT and ICS are highly sensitive and have been shown to detect CMV-specific memory T-cell responses in individuals lacking detectable serum antibodies, likely reflecting prior subclinical exposure, waning humoral immunity, or immune memory not captured by serology alone.” (Page 16, Lines 304 – 311).

Importantly, this study was designed as a comparative methodological analysis rather than a diagnostic study intended to define CMV serostatus, immune protection, or clinical cut-off values. We have clarified this scope in the revised Discussion and Limitations sections.

“Accordingly, this study should be interpreted as a comparative methodological evaluation of two established CMV-specific T-cell assays rather than a diagnostic study aimed at defining CMV serostatus, immune protection, or clinical cut-off values.” (Page 16, Lines 318 – 320).

**Minor points.**

1. At line 78, the authors posit that “humoral immune response could be crucial in restricting CMV dissemination.” This statement is, at a minimum, controversial, and requires adequate citation if it is to be included. Many would argue that humoral immunity is neither necessary nor sufficient to limit CMV dissemination.

We thank the reviewer for this important comment. We agree that the role of humoral immunity in restricting CMV dissemination remains controversial and that antibody responses alone are neither necessary nor sufficient for controlling CMV infection. To address this concern, we have revised the statement to soften the wording and to better reflect the current understanding that humoral immunity may contribute to CMV control in a supportive or complementary manner rather than being essential. We have also added appropriate citations to acknowledge the ongoing debate in the field (Page 4, Lines 78–81).

“Although cell-mediated immunity is considered the primary mechanism for controlling CMV infection, humoral immune responses may contribute to limiting viral dissemination in certain contexts, particularly by reducing viral spread and reinfection, but are not sufficient on their own.”

2. At line 88, the authors mention correlation between anti-CMV IgG and CMIR. Whether they indeed determined its relevance as a surrogate for CMIR is debatable, and given their lack of negative control, might be an overstatement of their findings.

We thank the reviewer for this comment. We agree that the relevance of anti-CMV IgG as a surrogate marker for CMIR cannot be established based on the current study design, particularly in the absence of a truly CMV-seronegative control group. Accordingly, we have revised the manuscript to avoid any implication that anti-CMV IgG serves as a surrogate for CMIR. The revised text now clearly states that the study explored the association between anti-CMV IgG levels and CMV-specific cellular immune responses in healthy individuals, and this relationship is presented as exploratory rather than confirmatory. (Page 4, Lines 89 –93).

“This study compared ELISPOT and intracellular cytokine staining (ICS) flow cytometry assays for the quantification of CMV-specific T-cell responses in peripheral blood mononuclear cells (PBMCs) from healthy individuals following CMV antigen stimulation. In addition, the study explored the association between anti-CMV IgG levels and CMV-specific cell-mediated immune responses (CMIR).”

3. Initially, I had some concerns about the presentation of flow cytometry data. However, the supporting document s1 shows a very nice example of how I wish others presented their gating strategies. Very nicely done, and thank you for this transparency.

We sincerely thank the reviewer for this positive feedback. We appreciate the reviewer’s recognition of this effort.

4. In the discussion, at line 242-243, the authors say that the correlation between ELISPOT and ICS “remains to be elucidated”. Yet, at lines 248-250, they cite at least two studies which do in fact show precisely that correlation. I’m confused, then, what aspect remains to be elucidated.

We thank the reviewer for this comment. We agree that correlations between ELISPOT and ICS have been demonstrated previously. The Discussion has been revised to clarify that our study does not claim novelty in establishing this correlation per se, but rather addresses the limited availability of direct comparative evaluations performed under identical experimental conditions. (Page 14, Lines 259 –261)

“Several previous studies have already demonstrated a significant correlation between these two assays; however, direct comparative evaluations under identical experimental conditions remain limited.”

5. At lines 286-292, the authors make several seemingly contradictory statements about CMIR and humoral immunity: the antibody response can act as a surrogate for CMIR; and yet, at the same time, CMIRs may be functional in the absence of a humoral response. It’s a relatively minor point – because perhaps there truly is value in the correlation, while in a subset of individuals the two metrics simply do not correlate. In its current presentation, it’s a bit confusing.

We thank the reviewer for highlighting this point. We have revised the Discussion to clarify that the apparent discrepancy reflects inter-individual variability rather than a contradiction between humoral and cellular immunity. The revised text emphasizes that CMV-specific T-cell responses can be detected in the absence of measurable antibodies and that our findings should be interpreted in the context of a methodological comparison rather than diagnostic inference. Regarding the use of antibody responses as a surrogate for CMIR, our findings suggest that the presence of an antibody response may serve as a predictor of CMIR. However, this relationship may not appear to be bidirectional. Specifically, the presence of CMIR does not consistently predict a measurable antibody response. Therefore, antibody response should be interpreted with caution when considered as a surrogate marker for CMIR. The following sentences were added. (Page 16, Lines 312-317)

“Though these findings suggest that the presence of an antibody response may serve as a predictor of CMIR, this relationship may not appear to be bidirectional. Specifically, the presence of CMIR does not consistently predict a measurable antibody response. Therefore, antibody response should be interpreted with caution when considered as a surrogate marker for CMIR, especially when the antibody response is absent.”

“In addition, this study used only IFN-γ-secreting/producing cells as the surrogate marker for CMV-specific T-cells, which can be detected in the absence of measurable antibodies and that our findings should be interpreted in the context of a methodological comparison rather than diagnostic inference.” (Page 17, Lines 332 – 335)

6. Finally, the authors should be careful in saying that the main limitation was that only healthy subjects were enrolled. It’s true that new information could be found in immunocompromised subjects, but I don’t consider that to be a shortcoming in this particular study. Instead, I’d prefer that they address the issue of no CMV-negative controls.

We thank the reviewer for this suggestion and agree that enrollment of healthy subjects should not be considered a major limitation for this methodological comparison. The Discussion has been revised to identify the lack of a clearly defined CMV-seronegative control group as the principal limitation of the study, which reflects the high CMV seroprevalence in our geographic region. Other limitations are now presented as secondary considerations. (Page 17, Lines 325 – 327)

“The main limitation of this study is the absence of a clearly defined CMV-seronegative control group, which is largely attributable to the very high CMV seroprevalence in the adult population in our geographic region.”

In addition, a previous study demonstrated that CMV seronegativity was found in only 2.4% (1 of 41 patients) of Thai young children who were evaluated before liver transplantation. (Reference: doi: 10.3390/v15112213)
