# Supplementary material for: Comparison between enzyme-linked immunospot assay and intracellular cytokine flow cytometry assay of cytomegalovirus-specific T-cell response in healthy participants
Source: PLoS One. 2026 Jun 4;21(6):e0349292. doi: 10.1371/journal.pone.0349292 (PMC13235872; doi:10.1371/journal.pone.0349292)
Supplement: Attachment — Submitted filename: Response_to_reviewers_auresp_2.docx [file pone.0349292.s005.docx]

**Response to Reviewers**

Manuscript ID: PONE-D-25-23529R1

Title: Comparison between enzyme-linked immunospot assay and intracellular cytokine flow cytometry assay of cytomegalovirus-specific T-cell response in healthy participants

Journal: PLOS ONE

Dear Dr. Sharma and the Reviewers,

We would like to sincerely thank the Academic Editor and the reviewers for their careful evaluation of our manuscript and for their constructive comments, which helped improve the quality and clarity of our work. We have carefully revised the manuscript in accordance with all suggestions. All changes have been incorporated into the revised manuscript and are highlighted in the tracked version.

Below, we provide a detailed, point-by-point response to each comment. Line numbers mentioned refer to the revised manuscript.

**Reviewer #3**

**Comment:**

All comments have been addressed.

**Response:**

We sincerely thank the reviewer for the positive evaluation of our revised manuscript and for the helpful suggestions provided in the previous review round, which significantly improved the manuscript.

**Reviewer #4**

**Comment:**

The study does not highlight novel findings since correlation between the methods is expected. It would be more interesting if immunosuppressed patients were included.

**Response:**

We thank the reviewer for this insightful comment. We agree that correlations between the ELISPOT assay and intracellular cytokine staining (ICS) for measuring CMV-specific T-cell responses have been reported in previous studies. The primary aim of the present study was not to establish a completely novel methodological relationship, but rather to provide an additional analytical comparison of these two widely used assays under the same experimental conditions and to further explore their relationship with CMV-specific humoral immunity. To better clarify the study's scientific positioning and contribution, we have revised the Introduction and Discussion sections.

First, in the Introduction, we added a paragraph emphasizing that although correlations between ELISPOT and ICS assays have been previously reported, comparative studies evaluating these two platforms alongside CMV-specific humoral immunity remain limited. We also highlighted that the relationship between CMV-specific cellular responses measured by these assays and anti-CMV IgG levels has not been well characterized in healthy individuals. (Lines 42 – 44, 92 - 96)

Second, in the Discussion, we expanded the interpretation of our findings to emphasize that our results provide additional analytical validation of the relationship between ELISPOT and ICS assays and extend previous observations by examining their association with CMV-specific antibody levels. (Lines 282 – 291)

Regarding the reviewer’s suggestion to include immunosuppressed patients, we agree that such populations would provide important clinical insights. However, the present study was designed as a methodological comparison using healthy individuals to minimize confounding factors related to immune dysfunction or immunosuppressive therapy. Future studies involving transplant recipients or other immunocompromised populations would be valuable for further evaluating the clinical utility of these assays for CMV immune monitoring.

**Comment 1:**

In Abstract: Lines 37, 38, and 39 contain unnecessary repetition about the seropositivity of the study participants.

**Response:**

We thank the reviewer for pointing out this issue. We have removed the redundant phrasing to improve clarity.

“Among the 30 participants, CMV-specific T cells were detected in all participants (100%), including the two seronegative individuals.” (Line 39 - 40)

**Comment 2:**

The last sentence in lines 42–43: “CMV IgG level could be a marker of past CMV infection and could be correlated with CMIR against CMV infection in immunocompetent hosts” is unclear in its meaning.

**Response:**

We thank the reviewer for pointing this out. The sentence has been revised to improve clarity. (Line 42 - 44)

“CMV IgG levels may reflect prior CMV infection and CMV-specific cell-mediated immunity (CMIR) in immunocompetent individuals.”

**Comment 3:**

In Introduction: Lines 49–50: The statement “Reactivation of CMV in healthy individuals is usually asymptomatic” is unclear and contradictory in wording.

**Response:**

We agree with the reviewer that the original phrasing was unclear. We have revised the statement to clarify that while CMV may undergo periodic reactivation without symptoms in immunocompetent individuals. The text has been revised. (Line 49 - 50)

“CMV reactivation typically remains asymptomatic in immunocompetent hosts, as robust immune surveillance prevents clinical disease.”

**Comment 4:**

Line 54–55 The sentence “Interferon-gamma (IFN-γ) secreted by these cells upon stimulation with specific antigens could represent that these cells have been activated [3–6]” is incorrect.

**Response:**

We thank the reviewer for pointing this out. The sentence has been revised to improve immunological accuracy. It now reads: Interferon-gamma (IFN-γ) production following stimulation with CMV antigens is commonly used as a functional readout of CMV-specific T-cell responses.

“Cell-mediated immune response (CMIR) plays a crucial role in controlling CMV replication. CD4⁺ T helper and CD8⁺ cytotoxic T lymphocytes are key mediators of CMV-specific immunity. Interferon-gamma (IFN-γ) production following stimulation with CMV antigens is commonly used as a functional readout of CMV-specific T-cell responses [3–6].” (Lines 52 – 56).

**Comment 5:**

Lines 58–59: The sentence “Nevertheless, other immunologically active cells also play a role in the defense against CMV infection” is also unclear, as it does not specify which cells are being referred to and lacks a reference; the subsequent sentence should be expressed more clearly. Unclear statement regarding other immunologically active cells.

**Response:**

We thank the reviewer for this comment. The sentence has been revised to clearly specify the immune cell types involved, and appropriate references have been added.

“In addition to T-lymphocytes, other immune cells also contribute to host defense against CMV infection. In particular, natural killer (NK) cells and natural killer T (NKT) cells have been reported to participate in the early immune response against CMV through cytotoxic activity and cytokine production.” (Lines 58 – 62).

**Comment 6:**

Lines 81–82: The sentence “Humoral immune response (HIR) could be crucial in restricting CMV dissemination and minimizing disease severity” requires a reference.

**Response:**

We thank the reviewer for this comment. The appropriate reference has been included.

Griffiths PD, Stanton A, McCarrell E, et al. Cytomegalovirus glycoprotein-B vaccine with MF59 adjuvant in transplant recipients: a phase 2 randomised placebo-controlled trial. Lancet. 2011;377(9773):1256-1263. doi:10.1016/S0140-6736(11)60136-0.

**Comment 7:**

Lines 86–88: The sentence “However, anti-CMV IgG may not provide overall immunological status against CMV infection, especially regarding CMIR” is inaccurate and requires a reference.

**Response:**

We sincerely thank the reviewer for this insightful comment. We agree that the original statement lacked precision. We have revised the text to clearly distinguish between serological status (anti-CMV IgG) and functional cell-mediated immune response (CMIR). The updated paragraph now emphasizes that while IgG indicates prior exposure, it does not necessarily reflect CMIR's protective capacity, which is the primary mechanism for controlling CMV latency and preventing reactivation. Relevant clinical and mechanistic references, including Jarque et al. (2020) and Crough & Khanna (2009), have been added to support this distinction.

“Notably, while anti-CMV IgG antibody levels correlate with the frequency of circulating CMV-specific memory B-cells [22] and serve as a reliable indicator of prior exposure, they do not directly reflect the functional vigor of the Cell-Mediated Immune Response (CMIR). Consequently, serological status alone is insufficient to assess comprehensive immunological protection, as T-cell-mediated pathways remain the cornerstone for controlling viral latency and preventing clinical reactivation [1,23]” (Lines 86 – 91).

**Comment 8:**

In Methods: The authors should describe how the individuals included in the study were selected.

**Response:**

We thank the reviewer for this comment. The inclusion and exclusion criteria have been added to the methods section. A total of 30 healthcare workers were recruited from Ramathibodi Hospital between November 7, 2023, and March 31, 2024. Participant selection was based on the following criteria:

- Inclusion Criteria:
  1. Aged 18–59 years.
  2. No history of autoimmune or immune-related diseases.
  3. No active viral infections.
  4. No chronic underlying diseases.
- Exclusion Criteria:
  1. Current or recent use of immunosuppressive drugs.
  2. Presence of immunocompromised conditions.

**Reviewer #5**

**Comment:**

The manuscript is technically sound and conclusions are supported by the data. No major concerns.

**Response:**

We thank the reviewer for the positive evaluation of our study and for recognizing the methodological rigor of our work.

**Reviewer #6**

**Major Comments**

**Comment 1:**

Novelty and scientific positioning need strengthening.

**Response:**

We thank the reviewer for this valuable comment. We agree that correlations between the ELISPOT assay and Intracellular Cytokine Staining for measuring T-cell responses to Cytomegalovirus have been previously reported. To better clarify the novelty and scientific positioning of our study, we have revised both the Introduction and Discussion sections.

First, in the Introduction, we have added a paragraph highlighting the existing gap in the literature. Specifically, we emphasized that comparative studies evaluating ELISPOT and ICS together with CMV-specific humoral immunity remain limited and that the relationship between CMV-specific cellular responses measured by these assays and anti-CMV IgG levels has not been fully characterized in healthy individuals. Lines 42 – 44 and 92 - 96.

Second, in the Discussion, we have added text to more clearly differentiate our findings from previous studies and to highlight the contribution of this work as an additional analytical validation of the relationship between ELISPOT and ICS assays in a cohort of healthy adults. We also expanded the discussion of the potential translational implications of our findings, particularly regarding the complementary roles of these assays and their practical use in CMV immune monitoring, depending on laboratory capacity and the level of cellular characterization required.

These revisions have been incorporated to better clarify the scientific contribution and practical relevance of the present study.

“Despite previous reports describing correlations between ELISPOT assay and Intracellular Cytokine Staining for the detection of CMV-specific T-cell responses to Cytomegalovirus, comparative data evaluating these two platforms together with CMV-specific humoral immunity remain limited. In particular, the relationship between CMV-specific T-cell responses measured by these assays and anti-CMV IgG levels has not been fully characterized in healthy individuals. Clarifying this relationship may help improve the understanding of the complementary roles of cellular and humoral immune responses and provide practical insights into assay selection for CMV immune monitoring. Therefore, this study aimed to provide additional analytical validation of the relationship between ELISPOT and ICS assays in a cohort of healthy adults and to further explore their association with CMV-specific antibody levels.” Lines 282 – 291.

“While previous studies have reported correlations between ELISPOT and ICS assays, our findings provide additional analytical validation of their relationship in a cohort of healthy adults and extend prior observations by exploring the association between CMV-specific cellular responses and CMV-specific humoral immunity.” Lines 301 – 304.

“From a practical perspective, ELISPOT offers a relatively simple and sensitive platform for detecting antigen-specific T-cell responses, whereas ICS allows detailed phenotypic characterization of responding T-cell subsets. The correlation observed between these two assays suggests that ELISPOT may serve as a practical alternative for CMV immune monitoring in laboratories where flow cytometry-based ICS is not routinely available.” Lines 320 –324.

“These findings further highlight the complementary roles of ELISPOT and ICS assays in CMV immune monitoring, depending on whether a laboratory prioritizes assay sensitivity or detailed cellular phenotyping.” Lines 335 – 337.

**Comment 2:**

Cohort selection limits clinical relevance.

**Response:**

We appreciate this important point. The manuscript has been revised to clarify that the study was designed as an analytical comparison in healthy immunocompetent individuals, and that the findings should be interpreted within this context. We now explicitly state that further validation in immunocompromised populations is required before clinical application (Lines 386 – 389).

**Comment 3:**

Correlation alone is insufficient to demonstrate agreement.

**Response:**

We thank the reviewer for this important suggestion. In response, we have performed an additional agreement analysis using the Bland–Altman analysis to further evaluate the agreement between the ELISPOT assay and Intracellular Cytokine Staining assays. This analysis allowed us to assess the systematic bias and limits of agreement between the two methods.

The analysis demonstrated relatively small mean biases but relatively wide limits of agreement, indicating variability between measurements obtained by the two assays. These findings suggest that although the assays show significant correlation, they should not be considered directly interchangeable for quantitative measurements.

The description of the Bland–Altman analysis and its results has now been added to the Results section (Lines 265 – 272), and the interpretation of these findings has been incorporated into the Discussion section (Lines 309 – 318).

**The Results section**

“To further evaluate the agreement between the ELISPOT assay and Intracellular Cytokine Staining, Bland–Altman analysis was performed. For stimulation with whole CMV lysate, the mean bias was 2,599 cells, with 95% limits of agreement from −13,338 to 18,535 cells. For stimulation with IE1, the mean bias between the two assays was 237 cells per 10⁶ CD3⁺ T cells, with 95% limits of agreement ranging from −3,676 to 4,151 cells. It should be noted that the results of the ELISPOT assay were expressed as spots per 10⁶ peripheral blood mononuclear cells (PBMCs), whereas ICS results were reported as cytokine-producing cells per 10⁶ CD3⁺ T-cells (Fig 5).”

**The Discussion section**

The Bland–Altman analysis demonstrated relatively small mean biases between the two assays; however, the wide limits of agreement indicate considerable variability between measurements obtained by the two methods. One possible explanation for this variability is the difference in the denominators used for quantification. The ELISPOT assay reports responses as spots per 10⁶ peripheral blood mononuclear cells, whereas ICS quantifies cytokine-producing cells per 10⁶ CD3⁺ T-cells.

Although both assays showed significant correlations and similar overall trends in detecting CMV-specific T-cell responses to Cytomegalovirus antigens, the relatively wide limits of agreement suggest that ELISPOT and ICS should not be considered directly interchangeable for quantitative measurements. Instead, these assays may provide complementary information for assessing CMV-specific cellular immune responses.

**Comment 4:**

Sample size and statistical power.

**Response:**

We thank the reviewer for this important comment. We agree that the relatively small sample size may influence the precision of the statistical estimates.

First, to improve the statistical reporting, we have now added 95% confidence intervals for all correlation coefficients in the Results section to provide additional information regarding the stability and precision of the estimated correlations.	Second, we acknowledge that the sample size of this study was relatively limited (n = 30). This cohort size was determined based on the availability of healthy volunteer samples and the exploratory nature of this study, which compares two cellular immune monitoring assays. Finally, we have expanded the Discussion section to more clearly acknowledge the limitations posed by the relatively small sample size and the high CMV seroprevalence in the study population, which may limit subgroup analyses and the generalizability of the findings. These revisions have been incorporated into the Results and Discussion sections of the revised manuscript.

“This study has several limitations. The primary limitation was the absence of a clearly defined CMV-seronegative control group, largely due to the high CMV seroprevalence in the adult population in our geographic region. This hindered our ability to perform meaningful subgroup analyses based on serostatus [37]. Furthermore, the sample size was relatively small (n = 30), which may limit the precision of the correlation estimates and agreement analyses. Larger studies would be required to confirm the robustness of these findings.” (Lines 381 – 386).

**Comment 5:**

 Interpretation of seronegative but T-cell–positive individuals

**Response:**

We thank the reviewer for this important comment. The two seronegative individuals exhibited a relatively high number of IFN-γ spot-forming units (SFU) detected by ELISPOT, while showing low frequencies of IFN-γ–producing T cells measured by ICS. This discrepancy was likely attributable to intrinsic differences between the two assays. ELISPOT measures cumulative IFN-γ secretion from all cell types within PBMCs immediately after stimulation, whereas ICS detects intracellular cytokine production within defined lymphocyte subsets following brefeldin A treatment. As a result, ELISPOT may capture IFN-γ secretion from non-T-cell populations or early secretory events that ICS does not detect.

This discordance may suggest that cellular immunity may persist even when antibody titers wane below the threshold of commercial detection. These seronegative/T-cell-positive cases indicate that T-cell IFN-γ production may be a more sensitive indicator of cumulative viral exposure in certain healthy individuals than traditional serology.

**Comment 6:**

Flow cytometry reporting requires additional detail

**Response:**

We thank the reviewer for this comment. The flow cytometry data were properly compensated during the initial analysis, and no changes to the compensation settings were applied. For clarification, we have included Figure 1 in this response to reviewers to illustrate the original compensation. The gating strategy for flow cytometry started with FSC and SSC singlets, with a total of 200,000 events acquired, as shown in Figure 2. Moreover, instrument QC procedures were performed daily before use. An example of the QC report is shown in Figure 3.

Figure 1. Flow cytometry compensation

Figure 2. The singlet gating strategy in flow cytometry

Figure 3. Cytometer Performance QC Report

**Comment 7:**

ELISPOT methodological detail

**Response:**

We thank the reviewer for raising this point. We acknowledge that ELISPOT assays are commonly performed in replicate wells; however, in this study, each condition was analyzed using a single well due to sample limitation and cell number constraints. To ensure data reliability despite the absence of technical replicates, several measures were implemented:

**Standardized assay conditions**: All samples were processed using the same protocol, reagents, and instrument settings to minimize technical variability.

**Spot counting criteria**: Spots were quantified using an automated ELISPOT reader with consistent predefined thresholds to reduce operator-dependent variability. Spot quality was checked using ImmunoSpot Software v7.0.26.0, with a minimum spot size of 0.001. Sq.mm and the maximum spot size is 0.0542 Sq.mm. Due to the high number of responses, wells exceeding 2000 spots were considered off-scale and reported accordingly. The off-scale values were found in the positive control. We acknowledge that accurate enumeration beyond this range is limited due to spot overlap and reduced counting precision.

**Data validation**: The results were interpreted in conjunction with complementary ICS assays, which demonstrated consistent trends.

**Minor Comments**

**Comment 1:**

NK cell frequency appears unusually low. The reported median NK cell proportion (~0.2%) is substantially below expected physiological ranges.

**Response:**

We sincerely thank the reviewer for raising this important and insightful point. We fully agree that natural killer (NK) cells typically account for approximately 10–20% of peripheral blood lymphocytes. The relatively lower proportion of NK cells observed in the present study is most likely attributable to a technical limitation related to the sample preparation protocol. Specifically, cells were fixed and permeabilized prior to antibody staining, a procedure that may alter antigenic epitopes and consequently reduce the sensitivity of CD56 antibody detection. This effect may have led to an underestimation of the NK cell population. In support of this explanation, we previously reported NK cell staining without formaldehyde fixation, in which the proportion of NK cells in the adult population was approximately 15%, consistent with the established literature. (Srisala S, et al. BMC Res Notes 2019; 12: 633). Since we did not compare the absolute number of NK cells between the 2 methods and all samples were treated with the same protocol, we believe that this would not greatly compromise the main results.

**Comment 2:**

 Blood processing and cell viability.

**Response:**

We thank the reviewer for this comment. Cell viability was assessed using Trypan blue staining. Viability was greater than 90% in all samples.

**Comment 3:**

 Statistical methods clarity

**Response:**

We sincerely thank the reviewer for raising this point and apologize for the oversight. Comparisons of CMV antigen responses between stimulated and non-stimulated groups were performed using the Wilcoxon signed-rank test. The strength of the correlations between the ICS assay and the ELISPOT assay, as well as between ICS or ELISPOT assays and anti-CMV IgG levels, was analyzed using Spearman’s rank correlation. The revised sentence has been added to the Results section.

“The Wilcoxon signed-rank test was also used to compare the frequencies of CMV-specific T-cells measured by ICS and ELISPOT assays.” (Lines 235 – 236).

**Comment 4:**

 Anti-CMV IgG handling: The manuscript states that values >250 AU/mL were truncated for analysis. Please justify this approach and discuss its potential impact on correlation estimates.

**Response:**

We thank the reviewer for this important observation regarding the quantification of high-titer samples. We chose to truncate values at the upper limit of quantification (250 AU/mL) rather than performing additional dilutions for the following reasons:

**Assay Validation and Standardization:** The commercial assay utilized in this study is analytically validated by the manufacturer for the range of 0 – 250 AU/mL. By remaining within this calibrated range, we ensured the highest level of precision and reproducibility.

**Biological Relevance:** The reactivity threshold is set at 6.6 AU/mL, a value of 250 AU/mL already represents a high-positive response (>40-fold the cutoff). In characterizing a healthy population (n=30), the primary objective was to distinguish between serostatus and the magnitude of the immune response within a standardized window; extrapolating outside this range may compromise the precision.

**Correlation Analysis**: To account for this truncation, we use Spearman’s analysis that relies on ranks rather than absolute magnitudes, it is robust to the truncation of values at the upper end of the scale, as all high-reactors are simply assigned a tied highest rank.

This criterion is based on the manufacturer’s recommendations and is consistent with previous studies (Eberhardt KA, Jung V, Knops E, et al. CMV-IgG pre-allogeneic hematopoietic stem cell transplantation and the risk for CMV reactivation and mortality. Bone Marrow Transplant. 2023;58(6):639-646. doi:10.1038/s41409-023-01944-2).

**Comment 5:**

 Figure presentation: adding regression lines with confidence intervals and harmonizing axis scales.

**Response:**

We thank the reviewer for this comment. Regression lines with 95% confidence intervals and harmonized axis scales have been added in figure 3.

**Comment 6:**

Language and style: The manuscript is generally clear but would benefit from minor English editing to improve flow and remove some redundancy in the Introduction and Discussion.

**Response:**

We thank the reviewer for this valuable suggestion. We have revised the manuscript to improve language clarity and flow throughout.
